# Does concurrent chemoradiotherapy preceded by chemotherapy improve survival in locally advanced nasopharyngeal cancer patients? Experience from Ghana

**DOI:** 10.1186/s41199-017-0023-3

**Published:** 2017-04-20

**Authors:** Joel Yarney, Naa A. Aryeetey, Alice Mensah, Emmanuel D. Kitcher, Verna Vanderpuye, Charles Aidoo, Kenneth Baidoo

**Affiliations:** 1 National Center for Radiotherapy and NuclearMedicine Korle bu Teaching, Hospital P.O. Box KB 369, Accra, Ghana; 2 School of Medicine and Dentistry, Accra, Ghana; 3 School of Biomedical and Allied Health Science University of Ghana, Accra, Ghana; 4 Department of Mathematics and Statistics, Accra Technical University, Accra, Ghana; 5 Ear Nose and Throat Unit, Department of Surgery, Accra, Ghana

**Keywords:** Neoadjuvant chemotherapy, Radiation, Nasopharyngeal cancer, Survival, Locally advanced disease

## Abstract

**Background:**

To find out how chemotherapy given prior to concurrent chemoradiotherapy compares with concurrent chemoradiation alone in the treatment of locally advanced nasopharyngeal cancer.

**Methods:**

Patient charts were examined and found to have submitted to one of two regimes as follows: Neoadjuvant chemotherapy consisting of Cisplatin and 5-fluorouracil followed by concurrent chemoradiotherapy with cisplatin (group1), or concurrent cisplatin based chemoradiotherapy only (group 2). Radiation treatment dose of 70Gy in 35 fractions was given in each group.

**Results:**

Forty-seven patients were evaluated with 68% male. Stage 4 disease comprised 83%, WHO type 3 was the commonest histologic type (53.2%). Median follow up period was 20 months (4–129). The 3-year overall survival for group 1 was 52.1%, and for group 2:65.7% (*p* = 0.47). The 3-year disease free survival for group 1 was 61.4, and 81.4% for group 2 (*p* = 0.03).

**Conclusion:**

The study revealed that concurrent chemoradiation alone yields better disease free survival compared to chemotherapy given prior to it. There is however no difference in overall survival between the two regimes.

## Background

Nasopharyngeal cancer is relatively uncommon worldwide, but shows distinct geographical and ethnic distribution [1]. It is endemic in Southeast Asia, where cured fish is a staple, the incidence is rising in North Africa [2]. It is the commonest head and neck malignancy at the Korle-Bu Teaching Hospital in Accra, Ghana, West Africa. The epidemiology of the disease has previously been described by the authors [3].

Infection with Epstein Barr virus (EBV) increases the risk of developing nasopharyngeal cancer in addition to genetic predisposition. EBV DNA and other nuclear components including nuclear antigens and viral encoded RNA(EBER) have been identified in tumor cells and plasma, providing opportunity for early detection and novel therapeutic approaches [4, 5].

The landmark paper published by Al- Saraf et al., that compared Cisplatin based concurrent chemoradiation with radiotherapy alone, established the former as the standard of care [6]. This finding has been validated by several studies and meta-analyses. In a meta-analysis of 8 randomized trials comparing radiotherapy to concurrent chemo-radiotherapy, concurrent chemo-radiotherapy resulted in overall survival benefit [7]. A previous meta-analysis had also confirmed concurrent chemo-radiotherapy as the most effective way of improving survival with a 20% improvement in overall survival at 5 years [8]. This finding was again replicated in a recent update to meta-analysis of chemotherapy in nasopharyngeal carcinoma collaborative group study (MAC-NPC) which showed no added benefit of adjuvant chemotherapy when concomitant chemoradiotherapy is used [9]. Induction chemotherapy followed by radiotherapy alone on the other hand has failed to demonstrate significant benefit [10]. Recent randomized studies have yielded conflicting outcomes when chemotherapy is given prior to concomitant chemoradiotherapy [11, 12].

The pattern of recurrence after concurrent chemoradiation alone for locally advanced disease which represents the norm in our setting [3], includes local, regional as well as distant failure, suggesting the need for novel therapeutic approaches to enable better disease control.

The authors previously reported 73.1% of patients presenting with stage IVB disease in Accra [3], this proportion of disease is much higher than in most series, and therefore presents unique challenges to treatment, indeed two thirds of all failures occurred loco-regionally. For these patients, rapid downsizing of disease which can be achieved with neoadjuvant chemotherapy is desirable.

The objectives of this study therefore were:

1. To describe the demography of nasopharyngeal cancer with respect to age, sex, and histological type.
2. To compare, the 3-year disease free and overall survival in patients treated with concurrent chemoradiotherapy only to those treated with neoadjuvant chemotherapy followed by concurrent chemoradiotherapy in locally advanced nasopharyngeal cancer patients.

## Methods

### Design

This is a single institution observational retrospective chart review.

### Inclusion criteria

All patients with histologically confirmed nasopharyngeal cancer referred for treatment were considered for chart review with the intention to select patient charts that meet the eligibility criteria for study. To be eligible for inclusion in the study, patients ought to have been treated with curative intent from January, 2000 to June, 2012, the end point for analysis was 31 December 2013. Eligible patients were required to have Eastern Cooperative Oncology Group (ECOG) performance status of at most 2. Patients ought to have adequate renal, liver and hematopoietic function.

### Exclusion criteria

Patients treated with palliative intent or for metastatic disease were excluded. Patients were not excluded from the study on the basis of toxicity.

### Staging

Patients were staged by physical and radiological examination using the 6^th^ edition of American Joint Cancer Committee on staging. Physical examination included fibre-optic endoscopy and biopsy under anesthesia with a description of the extent of disease. Staging work-up comprised Computed Tomography (CT) scan of the head and neck region, Chest X ray (CXR) and ultrasound of the abdomen and pelvis, and bone scintigraphy where indicated. CT scan: chest, abdomen and pelvis was performed only when CXR and ultrasound of the abdomen were equivocal.

### Treatment

#### Chemotherapy

Neoadjuvant chemotherapy consisted of 3 weekly Cisplatin at 80 mg/m^2^ on day 1 with 5-fluorouracil at 1000 mg/m^2^on day 1–4 or Capecitabine at 1000 mg/m^2^ twice a day for fourteen days (only two patients), 3 weekly for 2–3 cycles followed by chemo- radiation. Patients received only Cisplatin at a dose of 100 mg/m^2^ during radiotherapy on days 1,22 and 43 of radiotherapy.

### Radiation

All patients were immobilized in a Med Tec mask, and simulated with a conventional simulator after submitting to dental assessment. Some patients underwent hearing test as well. Patients received external beam radiation using 2-D planning as follows: Treatment fields consisted of two lateral opposed fields matched with a supraclavicular field, with appropriate shielding and using shrinking field technique. A posterior neck field was placed with a central block to shield the spinal cord whilst treating the involved nodes to the prescribed dose.

Patients were prescribed to receive:70Gy in 35 fractions at 2 Gy per fraction per day to the mid plane over seven weeks to the primary and involved nodes. Uninvolved neck was treated to 50Gy using similar fractionation schedule.

Megavoltage verification or port films were obtained of all fields prior to, and mid-way through treatment, and localization adjusted accordingly. The institution participates in the International Atomic Energy Agency postal audits on a yearly basis and records figures within 5% accuracy level.

### Toxicity

Patients were reviewed once a week during treatment to determine toxicity with weekly complete blood count, urea, electrolytes and creatinine. Acute and late toxicities were gleaned from the patient chart as recorded by treating Physician. Only grade 3 and 4 toxicities in eligible patients were assessed. A feeding tube was placed when required.

### Follow up

Following completion of treatment, patients were reviewed by Oncologists and Otolaryngologists three monthly for the first 2 years, and at most 6 monthly thereafter with clinical examination including fibre-optic examination.

Re-staging CT scan was ordered based on clinical suspicion of recurrence with biopsy where indicated. The date of death was ascertained by making telephone calls to next of kin or from death certificates.

### Statistics

Patient age, sex, histology, primary tumor stage T, and nodal stage N, were extracted from the charts and analyzed for summary statistics using Statistical Package for Social Science version 16 software, with description of mean, frequency, range and standard deviation where appropriate. Log-rank test was performed to determine differences in overall and disease free survival on the Kaplan Meier curve. Probability of < 0.05 was chosen as the level of significance.

### Limitations

The study is limited by the small number of patients and its retrospective nature.

## Results

Ninety-nine patients were identified to have been referred with a histologically confirmed diagnosis of nasopharyngeal cancer between January 2000 and June 2012, to the Radiotherapy Department, Korle-Bu Teaching Hospital in Accra, Ghana. Out of this number, only forty-seven met the inclusion criteria, the rest were excluded on the following basis: twenty-eight were excluded because they were treated with palliative intent, nine received radiation only, four were non-Ghanaian, left the country after treatment, and therefore had no follow- up data, and eleven were excluded because they absconded treatment before two weeks into it.

The mean age was 34.1 years with a range of 10–83 years. Only one patient was in the pediatric age bracket of twelve years or less. The commonest histological type going by WHO classification was type 3 (53.2%), followed by type 1 (25.5%), the least common was type 2(14. 9%), unknown comprised 6.4%. Male to female ratio was 2.1: 1.

Patients with Stage 4 disease comprised 83% (38.3% in group 1 and 44.7% in group 2). Staging characteristics is shown in Table 1.

**Table 1:** Staging characteristics

Stage						
T-Stage	N-Stage					Total
	0	1	2	3	X	
1	0	0	1	2	0	3
2	0	1	1	7	0	9
3	0	5	2	8	0	15
4	4	2	6	7	1	20
Total	4	8	10	24	1	47

Patients who fulfilled the inclusion criteria were identified to have been treated with either of two regimes. Group 1 received neoadjuvant chemotherapy followed by concurrent chemoradiation, they were 22 in number; Group 2 patients received chemoradiation only and comprised 25 patients. Characteristics of patients in each group is shown in Table 2, other than age, there was no statistical difference between the two groups in the parameters examined.

**Table 2:** Group characteristics

	Group 1	Group 2	*P* – Value @ 95% CI
Toxicity			
Neutropenia	8	5	
Trismus	5	9	
Age (years)			
< 20	15	8	0.013
> 20	7	17	
Sex			
Male	17	15	0.205
Female	5	10	
Stage			
I	–	1	0.422
III	3	3	
IV	18	21	
Unknown	1	–	
Who type			
1	5	7	0.214
2	4	3	
3	13	12	
Not stated	–	3	

Two patients in group 2 had persistent neck disease at the end of treatment, 2 more in this group failed in the neck during the follow up period. Four patients had persistent disease in the neck in group 1. There were five distant events including one in the liver and lung, and three in bone for group one. Group 2 patients had two failures in bone. Six and three patients suffered recurrence in the nasopharynx in groups 1 and 2 respectively. Some of the recurrences were concurrent events.

**Fig. 1:**
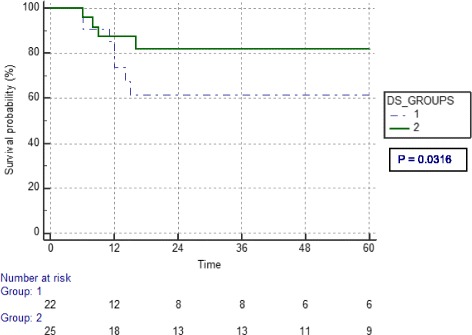
Kaplan Meier curve of disease free survival in months

**Fig. 2:**
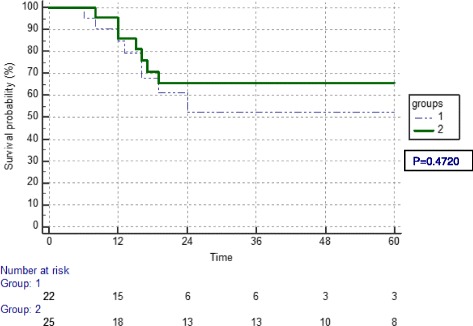
Kaplan Meier curve of overall survival in months

The median follow-up period was 20 months (4–129). The three-year disease free survival rates for groups one and two were 61.4 and 81.4% respectively yielding a *p* value of 0.03 on log rank test. The three-year overall survival for groups one and two were 52.1 and 65.7% respectively, yielding a *p* value of 0.47. Kaplan Meier curves for disease free and overall survival are shown in Figs. 1 and 2 respectively. More patients in Group 1, 8 in all, developed neutropenia, compared to 5 in Group 2. Nine patients in Group 2 developed trismus compared to 5 in Group 1. Average number of chemotherapy cycles received during concurrent chemoradiotherapy was not significantly different between the two groups.

## Discussion

Head and Neck cancer is the third commonest malignancy seen at the Radiotherapy Centre in Accra, and nasopharyngeal cancer is the commonest amongst them. This observation probably reflects low incidence of smoking in Ghana relative to the developed world where laryngeal cancer is more common.

The observed incidence of nasopharyngeal cancer is probably due to consumption of salt cured fish which is a delicacy, EBV infection, as well as genetic predisposition. Epstein Bar viral antigen was not tested in this chart review.

Concurrent chemoradiation has been established as the standard of care in the management of nasopharyngeal cancer, all patients therefore submitted to concurrent chemoradiation, it was however left to the discretion of the treating Physician to determine whether this would be preceded by neoadjuvant chemotherapy as it holds the promise of down-sizing tumors especially with extracted evidence from management of other head and neck cancer. The safety and efficacy of induction chemotherapy followed by concomitant chemoradiation was reported by Al-Amro et al. in 2005 [13], in addition, adjuvant chemotherapy has not been shown to impact on overall survival [7, 8, 14].

The observed male to female ratio is similar to previous reports. World Health Organization (WHO) type 3 is the commonest variety, consistent with the picture in endemic regions, followed by type 1.

Following the study by Al Saraf et al. that included patients with predominantly non-endemic variety, concerns were expressed regarding the applicability of the findings of that study to endemic regions. Subsequent studies have however validated the conclusion of that study in different geographical settings with varying distribution of WHO type, thus establishing concurrent chemoradiotherapy as the standard of care in all geographical regions [7–9].

The ten-year-old patient was treated with the Al Saraf protocol because of the presence of extensive disease at presentation. This was based on the recommended dose range of 50–72 Gy in patients 10 years and older, this dose is reduced by 5–10% in children under 10 years [15–17]. The other patients were twelve years or older.

Owing to the limited number of patients studied, the number of failures were also small. From the results on recurrence pattern: the number of regional failures were the same for both groups. There were more failures in the nasopharynx and distantly for group 1 relative to group 2, we are however unable to comment on the statistical significance of this observation owing to the small numbers.

Chen YP et al. reported that neoadjuvant chemotherapy followed by concurrent chemoradiotherapy is associated with reduced distant failures, as compared with concurrent chemoradiotherapy alone and whether the addition of neoadjuvant chemotherapy can improve survival for locoregionally advanced nasopharyngeal cancer should be further explored. Optimizing regimes and identifying patients at high-risk of metastases may enhance the efficacy of neoadjuvant chemotherapy followed by concurrent chemoradiotherapy [18].

In a study from South Korea, 300 patients were selected after matching for analysis. Higher 5-year locoregional failure free survival was observed in the chemoradiotherapy only arm (85% vs 72%, *p* = 0.014). No significant difference in distant failure-free survival (DFFS), disease- free survival (DFS), and overall survival were observed between the groups. In subgroup analysis, the neoadjuvant chemotherapy arm showed superior DFFS and DFS in stage IV patients younger than 60 years. No significant difference in compliance and toxicity was observed between groups, except the radiation therapy duration was slightly shorter in the concurrent chemoradiotherapy only arm. The authors concluded that the study did not show superiority of neoadjuvant chemotherapy followed by concurrent chemoradiotherapy over concurrent chemoradiotherapy alone. Because neoadjuvant chemotherapy could increase the risk of locoregional recurrence, it can only be considered in selected young patients with advanced stage IV disease. The role of neoadjuvant chemotherapy remains to be defined and should not be viewed as the standard of care [19]. Even though small, our study seems to support findings from other studies that neoadjuvant chemotherapy patients appear to have more local failures.

Our 3-year overall survival of 65.7%, and disease free survival of 81.4% in the concurrent chemoradiation only group is modest considering the high proportion of Stage 4 disease.

Comparing the overall survival between the two groups on log-rank test, we were unable to detect a statistically significant difference between the two groups, *p* = 0.47. There was however a statistically significant difference between the two groups in relation to disease free survival in favor of chemoradiation only, with a 3-year value of 81.4% in the chemoradiation only group, versus 61.4% in the neoadjuvant followed by chemoradiation group, *p* = 0.03 on log-rank test.

It is evident from other studies that even though neoadjuvant chemotherapy can achieve substantial tumor downsizing, it does not translate into improved disease free or overall survival [14]. In this study, neoadjuvant chemotherapy was found to be inferior to chemoradiation only, in terms of disease free survival.

Fountzilas G et al. [11] demonstrated that induction chemotherapy with three cycles of cisplatin, epirubicin and taxol followed by concomitant chemoradiation did not significantly improve response rates, progression free or overall survival relative to concomitant chemoradiation alone; 3- year overall survival was quoted as 66.6 and 71.8% respectively (*p* = 0.652). Contrary to the afore mentioned trial, Hui EP et al. [12] in a phase II trial, reported 3- year overall survival of 94.1 and 67.7% with a *p* value of 0.012 in favor of neoadjuvant chemotherapy. A recent trial by Sun Y and colleagues demonstrated a 3- year failure free survival of 80% in the group that received neoadjuvant chemotherapy using cisplatin, docetaxel and 5- fluorouracil followed by concurrent chemoradiation, relative to 72% in the group that received concurrent chemoradiation alone, *p* = 0.034, but with more toxicity [20].

There are similar studies that seem to suggest that upfront chemotherapy followed by concomitant chemoradiotherapy has high efficacy, demonstrated by progression free and overall survival, but these outcomes were not compared to concomitant chemoradiation alone [18, 21].

Neoadjuvant chemotherapy followed by radiotherapy may significantly reduce the risk of locoregional recurrence and distant metastases and may improve disease specific survival in locally advanced nasopharyngeal cancer as demonstrated by Chua et al. [10], and therefore has a theoretical potential to confer improvement in overall survival, it must however be noted that in that study it was compared to radiotherapy alone. In a phase 2 study involving neoadjuvant chemotherapy followed by concurrent chemoradiation by Kong L et al., docetaxel was given in addition to cisplatin and 5-fluorouracil prior to concurrent cisplatin based chemoradiation and a 3- year overall survival of 90.2% was reported in patients with stage IVA/IVB disease, thus demonstrating the potential high efficacy of this maneuver [22].

Despite the above, an interim analysis of a phase 3 study in which patients received three cycles of a 3-weekly regimen that employed relatively newer agents viz gemcitabine, carboplatin and paclitaxel prior to cisplatin based concurrent chemoradiation compared to concurrent chemoradiation alone was reported early because it had crossed the statistical boundary for futility in the use of neoadjuvant chemotherapy. There was no statistically significant difference between the two arms for overall survival, disease free survival and distant metastases free survival [23]. There was however considerably higher hematological toxicity and fatigue in the neoadjuvant chemotherapy group even though global quality of life scores were identical.

A recent study from Taiwan comparing concurrent chemoradiotherapy to neoadjuvant chemotherapy followed by radiation alone also failed to show superiority of neoadjuvant chemotherapy, reporting similar 5-year overall survival, indeed among patients who were recurrence-free in the first 2 years after treatment, those treated with neoadjuvant chemotherapy experienced poorer locoregional control that reached statistical significance [24]. It is believed that neoadjuvant chemotherapy leads to selection of resistant cell, accelerated repopulation and reduced compliance to chemoradiation, perhaps this deficiency is offset by concurrent chemoradiation more efficiently than radiation alone.

Toxicity reporting in our study was inadequate.

Modern radiotherapy is performed using Intensity Modulated Radiation Therapy (IMRT) or Volume Modulated Arc Therapy (VMAT). We also acknowledge that there could be stage migration if the patients had been staged with Magnetic Resonance Imaging (MRI); this imaging modality can also improve tumor localization during target volume delineation and therefore afford better tumor control. Despite these deficiencies, we believe that both groups were subjected to the same staging procedures, and treatment, and therefore any difference in outcomes is attributable to the treatment they received and not the sophistication of the treatment received.

## Conclusion

The epidemiology of nasopharyngeal cancer in our setting is similar to endemic regions with a predominance of the type 3 variety. Neoadjuvant chemotherapy followed by concurrent chemoradiation did not improve disease free or overall survival compared to concurrent chemoradiation alone in this group of patients. Neoadjuvant chemotherapy appears to be associated with more hematological toxicity, and may be responsible for more failures in the nasopharynx.

## Abbreviations

CT scan: Computed tomography scan
CXR: Chest X Ray
DNA: Deoxy ribonucleic acid
EBER: Epstein barr encoded RNA
EBV: Epstein barr virus
ECOG: Eastern cooperative oncology group
IMRT: Intensity modulated radiation therapy
VMAT: Volume modulated arc therapy
WHO: World health organization

## References

[1] Torre LA, Bray F, Siegel RL, Ferlay J, Lortet- Tieulent J, Jemal A. Global Cancer Statistics 2012 CA. Cancer J Clin. 2015;65:87–108. 10.3322/caac.21262 25651787

[2] Wee J, Tan EH, Tai BC, Wong HB, Leong SS, Tan T (2005). Randomized trial of radiotherapy versus concurrent chemoradiotherapy followed by adjuvant chemotherapy in patients with American Joint Committee on Cancer/International Union against cancer stage III and IV nasopharyngeal cancer of the endemic variety. J Clin Oncol.

[3] Yarney J, Vanderpuye V, Kitcher ED (2008). Treatment outcome of locally advanced nasopharyngeal cancer with concurrent chemoradiotherapy. West Afr J Med.

[4] Chan KC, Hung EC, Woo JK, Chan PK, Leung SF, Lai FP (2013). Early detection of nasopharyngeal carcinoma by plasma Epstein Barr virus DNA analysis in a surveillance programme. Cancer.

[5] Taylor GS, Jia H, Harrington K, Lee LW, Turner J, Ladell K (2014). A recombinant modified vaccinia Ankara vaccine encoding Epstein- Barr virus targeted antigens: a phase 1 trial in UK patients with EBV-positive cancer. Clin Cancer Res.

[6] Al-Sarraf M, LeBlanc M, Giri PG, Fu KK, Cooper J, Vuong T (1998). Chemoradiotherapy versus radiotherapy in patients with advanced nasopharyngeal cancer: phase III randomized Intergroup study 0099. J Clin Oncol.

[7] Baujat B, Audry H, Bourhis J, Chan AT, Onat H, Chua DT (2006). Chemotherapy in locally advanced nasopharyngeal carcinoma: an individual patient data meta-analysis of eight randomized trials and 1753 patients. Int J Radiat Oncol Biol Phys.

[8] Langendijk JA, Leemans CR, Buter J, Berkhof J, Slotman BJ (2004). The additional value of chemotherapy to radiotherapy in locally advanced nasopharyngeal carcinoma: a meta-analysis of the published literature. J Clin Oncol.

[9] Blanchard P, Lee A, Marguet S, Leclercq J, Ng WT, Ma J (2015). Chemotherapy and radiotherapy in nasopharyngeal carcinoma: an update of the MAC-NPC meta-analysis. Lancet Oncol.

[10] Chua DT, Ma J, Sham JS, Mai HQ, Choy DT, Hong MH (2005). Long- term survival after cisplatin-based induction chemotherapy and radiotherapy for nasopharyngeal carcinoma: a pooled data analysis of two phase III trials. J Clin Oncol.

[11] Fountzilas G, Ciuleanu E, Bobos M, Kalogera-Fountzila A, Eleftheraki AG, Karayannopoulou G (2012). Induction chemotherapy followed by concomitant radiotherapy and weekly cisplatin versus the same concomitant chemoradiotherapy in patients with nasopharyngeal carcinoma: a randomized phase II study conducted by the Hellenic Cooperative Oncology Group (HeCOG) with biomarker evaluation. Ann Oncol.

[12] Hui EP, Ma BB, Leung SF, King AD, Mo F, Kam MK (2009). Randomized phase II trial of concurrent cisplatin-radiotherapy with or without neoadjuvant docetaxel and cisplatin in advanced nasopharyngeal carcinoma. J Clin Oncol.

[13] Al- Amro A, Al Rajhi N, Khfaga Y, Memon M, Al Hebshi A, El-Enbabi A (2005). Neoadjuvant chemotherapy followed by concurrent chemo-radiation in locally advanced nasopharyngeal cancer. Int J Rad Oncol.

[14] Chen L, Hu CS, Chen XZ, Hu GQ, Cheng ZB, Sun Y (2012). Concurrent chemoradiotherapy plus adjuvant chemotherapy versus concurrent chemotherapy alone in patients with locoregionally advanced nasopharyngeal carcinoma: a phase 3 multi-centre randomized controlled trial. Lancet Oncol.

[15] Kao WC, Chen J-S, Yen C-J (2016). Advanced nasopharyngeal carcinoma in children. J Cancer Res Pract.

[16] Liu W, Tang Y, Gao L, Huang X, Luo J, Zhang S (2014). Nasopharyngeal carcinoma in children and adolescents-a single institution experience of 158 patients Radiation. Oncology.

[17] Ayan I, Kaytan E, Ayan N (2003). Childhood nasopharyngeal carcinoma: from biology to treatment. Lancet Oncol.

[18] Chen YP, Guo R, Liu N, Liu X, Mao YP, Tang L-L (2015). Efficacy of the additional neoadjuvant chemotherapy to concurrent chemoradiotherapy for patients with locoregionally advanced nasopharyngeal carcinoma: a Bayesian network meta- analysis of randomized controlled trials. J Cancer.

[19] Song JH, Wu H-G, Keam BS, Han JH, Ahn YC, Oh D (2016). The role of neoadjuvant chemotherapy in the treatment of nasopharyngeal carcinoma: a multi-institutional retrospective study (KROG 11–06) using propensity score matching analysis. Cancer Res Treat.

[20] Sun Y, Li W-F, Chen N-Y, Zhang N, Hu G-Q, Xie F-Y (2016). Induction chemotherapy plus concurrent chemotherapy versus concurrent chemotherapy alone in locoregionally advanced nasopharyngeal carcinoma: a phase 3, multicenter randomized controlled trial. Lancet Oncol.

[21] Golden DW, Rudra S, Witt ME, Nwizu T, Cohen EEW, Blair E (2013). Outcomes of induction chemotherapy followed by concurrent chemoradiation for nasopharyngeal cancer. Oral Oncol.

[22] Kong L, Hu C, Niu X, Zhang Y, Guo Y, Tham IW (2013). Neoadjuvant chemotherapy followed by concurrent chemoradiation for locally advanced nasopharyngeal carcinoma: Interim results from two prospective phase 2 clinical trials. Cancer.

[23] Tan T, Lim WT, Fong KW, Cheah SL, Soong YL, Ang MK et al. Randomized phase III trial of concurrent chemoradiation with or without neoadjuvant gemcitabine, carboplatin, and paclitaxel in locally advanced nasopharyngeal cancer. J ClinOncol 32:5 s, 2014(suppl; abstr 6003).

[24] Wu SY, Wu YH, Yang MW, Hsueh WT, Hsiao JR, Tsai ST (2014). Comparison of concurrent chemoradiotherapy versus neoadjuvant chemotherapy followed by radiation in patients with advanced nasopharyngeal carcinoma in endemic area: experience of 128 consecutive cases with 5-year follow-up. BMC Cancer.

